# Draft Metagenome-Assembled Genome Sequence of a Novel *Citricoccus* Species from Agricultural Soil in Western Colorado

**DOI:** 10.1128/mra.00359-22

**Published:** 2023-01-04

**Authors:** Paul O’Toole, Rebecca A. Daly, Reza Keshavarz Afshar, Michael Shaffer, Kelly C. Wrighton, Bridget B. McGivern

**Affiliations:** a Department of Microbiology, Colorado State University, Fort Collins, CO, USA; b Department of Soil and Crop Science, Colorado State University, Fort Collins, CO, USA; c Western Colorado Research Center, Fruita, CO, USA; University of Rochester School of Medicine and Dentistry

## Abstract

Members of the genus *Citricoccus* are recognized as salt-tolerant soil microorganisms. Here, we report the metagenome-assembled genome sequence of a novel *Citricoccus* species recovered from untilled, surface agricultural soils in western Colorado.

## ANNOUNCEMENT

The genus *Citricoccus* was first described after a bacterium was isolated from a mural in Austria (1). Physiologically characterized members of this genus are aerobic Gram-positive cocci that persist in diverse environmental conditions, with notable halotolerance (2–4), likely making them well-suited to growth in arid soils, where salinity is a common problem (5). Here, we present a draft metagenome-assembled genome (MAG) sequence representing a potential new species from the genus *Citricoccus*, which was recovered from agricultural soil.

Soil samples were taken from the Western Colorado Research Center (WCRC) in Fruita, CO, on 10 February 2021 at soil depths ranging from 0 to 5 cm. These soils were managed under a no-till, furrow-irrigated production system since 2017. At the time of sampling, fields were in a seasonal fallow following corn harvest in November. DNA was extracted from 0.4 g of soil using the Zymo quick-DNA fecal/soil microbe microprep kit (Zymo, Irvine, CA), following the “soil” protocol. Metagenomic libraries were prepared using the Tecan Ovation ultralow system v2, following kit protocols for input DNA fragmentation, and were sequenced on the NovaSEQ6000 platform on an S4 flow cell at Genomics Shared Resource, Colorado Cancer Center, Denver, CO. We obtained 37.9 Gbp of 150-bp paired-end reads that we assembled to recover MAGs. Read quality control was conducted using FastQC (v0.11.2) and trimmed using sickle (v1.33; pe -t sanger) (6). The resulting 37,589,610,518 trimmed reads were assembled using MEGAHIT (v1.2.9) (7) and were binned using MetaBat2 (v2.12.1, –k-min 31 –k-max 121 –k-step 10 –mem-flag 1) (8). Metagenome-assembled genome (MAG) quality was assessed using CheckM (v1.1.2) (9), and taxonomy was assigned using the Genome Taxonomy Database toolkit (GTDB-tk; v1.5.0, r202) (10). MAG annotation was performed using DRAM (11) within KBase (12, 13). Default parameters were used unless noted.

**FIG 1:**
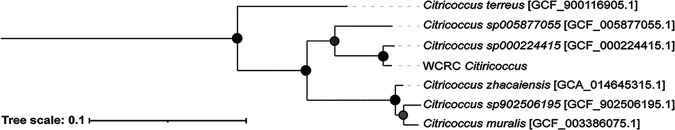
Phylogenetic tree based on the WCRC *Citricoccus* MAG and GTDB-tk r202 *Citricoccus* species representatives. The tree is rooted on the species representatives of g__Micrococcus. The GTDB-tk de_novo_wf workflow was used to generate a multiple sequence alignment (MSA) using g__Micrococcus as the outgroup and filtering to g__Citricoccus. The resulting MSA was used to construct a maximum-likelihood phylogenetic tree using RaxML (v8.2.9) (15) using the PROTGAMMAWAG model and 100 bootstraps. Bootstraps are colored gray for those greater than 88% (*n* = 2) and black for those = 100% (*n* = 4).

The MAG was assigned as a new *Citricoccus* species by GTDB-tk, joining eight *Citricoccus* genomes across six species (GTDB-tk r202). The MAG is most closely related to *Citricoccus sp000224415* by genome amino acid identity (92.5%) (14) and marker gene phylogeny (Fig. 1). The genome statistics for the WCRC *Citricoccus* MAG are given in Table 1.

**TABLE 1:** Metagenome-assembled genome statistics for WCRC *Citricoccus*

Parameter	Data
BioSample no.	SAMN26177294
Genome size (bp)	3,394,226
No. of contigs	433
GC content (%)	71.0
Longest contig (bp)	33,106
*N*_50_ value (bp)	9,996
Completeness (%)	91.02
Contamination (%)	2.24
No. of predicted coding genes	3,729
No. of tRNAs	47
Encoded rRNA	5S
Mean base coverage (×)	21.32

Annotation of the *Citricoccus* MAG indicated that this MAG is likely a facultative aerobe, with the ability to use oxygen and with an encoding potential for nitrate and nitrite reduction. Notably, characterization of Citricoccus nitrophenolicus (4), Citricoccus muralis (1), and Citricoccus zhacaiensis (2) indicated these species could not grow facultatively with nitrate or nitrite, and genomic analysis revealed they did not carry these genes. Therefore, nitrate and nitrite respiration may be unique to this new *Citricoccus* species. This MAG encodes the potential for the synthesis and transport of the osmolyte ectoine via an ectoine synthase and the ectoine/hydroxyectoine ABC transporter system (EhuABCD), respectively, possibly supporting the survival of this organism in arid, saline agricultural soils.

### Data availability.

The sequencing data for this project are deposited under BioProject number PRJNA725542. The MAG is deposited under BioSample SAMN26177294. The KBase narrative for the MAG annotation is available (13). The metagenomic reads were deposited in the Sequence Read Archive under accession SRS11831377.

## References

[1] Altenburger P, Kämpfer P, Schumann P, Steiner R, Lubitz W, Busse HJ. 2002. *Citricoccus muralis* gen. nov., sp. nov., a novel actinobacterium isolated from a medieval wall painting. Int J Syst Evol Microbiol 52:2095–2100. doi:10.1099/00207713-52-6-2095. 12508874

[2] Meng F-X, Yang X-C, Yu P-S, Pan J-M, Wang C-S, Xu X-W, Wu M. 2010. *Citricoccus zhacaiensis* sp. nov., isolated from a bioreactor for saline wastewater treatment. Int J Syst Evol Microbiol 60:495–499. doi:10.1099/ijs.0.011635-0. 19654363

[3] Li W-J, Chen H-H, Zhang Y-Q, Kim C-J, Park D-J, Lee J-C, Xu L-H, Jiang C-L. 2005. *Citricoccus alkalitolerans* sp. nov., a novel actinobacterium isolated from a desert soil in Egypt. Int J Syst Evol Microbiol 55:87–90. doi:10.1099/ijs.0.63237-0. 15653858

[4] Nielsen MB, Kjeldsen KU, Ingvorsen K. 2011. Description of *Citricoccus nitrophenolicus* sp. nov., a para-nitrophenol degrading actinobacterium isolated from a wastewater treatment plant and emended description of the genus Citricoccus Altenburger et al. 2002. Antonie Van Leeuwenhoek 99:489–499. doi:10.1007/s10482-010-9513-6. 20882410

[5] Jordán MM, Navarro-Pedreño J, García-Sánchez E, Mateu J, Juan P. 2004. Spatial dynamics of soil salinity under arid and semi-arid conditions: geological and environmental implications. Environ Geol 45:448–456. doi:10.1007/s00254-003-0894-y.

[6] Joshi N, Fass J. 2011. Sickle: a sliding-window, adaptive, quality-based trimming tool for FastQ files (Version 1.33) [Software]. https://github.com/najoshi/sickle. Retrieved 2 July 2020.

[7] Li D, Liu C-M, Luo R, Sadakane K, Lam T-W. 2015. MEGAHIT: an ultra-fast single-node solution for large and complex metagenomics assembly via succinct de Bruijn graph. Bioinformatics 31:1674–1676. doi:10.1093/bioinformatics/btv033. 25609793

[8] Kang DD, Li F, Kirton E, Thomas A, Egan R, An H, Wang Z. 2019. MetaBAT 2: an adaptive binning algorithm for robust and efficient genome reconstruction from metagenome assemblies. PeerJ 7:e7359. doi:10.7717/peerj.7359. 31388474 PMC6662567

[9] Parks DH, Imelfort M, Skennerton CT, Hugenholtz P, Tyson GW. 2015. CheckM: assessing the quality of microbial genomes recovered from isolates, single cells, and metagenomes. Genome Res 25:1043–1055. doi:10.1101/gr.186072.114. 25977477 PMC4484387

[10] Chaumeil PA, Mussig AJ, Hugenholtz P, Parks DH. 2019. GTDB-Tk: a toolkit to classify genomes with the genome taxonomy database. Bioinformatics 36:1925–1927. doi:10.1093/bioinformatics/btz848. 31730192 PMC7703759

[11] Shaffer M, Borton MA, McGivern BB, Zayed AA, La Rosa SL, Solden LM, Liu P, Narrowe AB, Rodríguez-Ramos J, Bolduc B, Gazitúa MC, Daly RA, Smith GJ, Vik DR, Pope PB, Sullivan MB, Roux S, Wrighton KC. 2020. DRAM for distilling microbial metabolism to automate the curation of microbiome function. Nucleic Acids Res 48:8883–8900. doi:10.1093/nar/gkaa621. 32766782 PMC7498326

[12] Arkin AP, Cottingham RW, Henry CS, Harris NL, Stevens RL, Maslov S, Dehal P, Ware D, Perez F, Canon S, Sneddon MW, Henderson ML, Riehl WJ, Murphy-Olson D, Chan SY, Kamimura RT, Kumari S, Drake MM, Brettin TS, Glass EM, Chivian D, Gunter D, Weston DJ, Allen BH, Baumohl J, Best AA, Bowen B, Brenner SE, Bun CC, Chandonia J-M, Chia J-M, Colasanti R, Conrad N, Davis JJ, Davison BH, DeJongh M, Devoid S, Dietrich E, Dubchak I, Edirisinghe JN, Fang G, Faria JP, Frybarger PM, Gerlach W, Gerstein M, Greiner A, Gurtowski J, Haun HL, He F, Jain R, et al. 2018. KBase: the United States Department of Energy Systems Biology Knowledgebase. Nat Biotechnol 36:566–569. doi:10.1038/nbt.4163. 29979655 PMC6870991

[13] McGivern BB, O’Toole P, Wrighton KC. 2022. O’Toole et al Citricoccus MRA genome announcement analysis narrative. doi:10.25982/130581.14/1897514.

[14] The enveomics collection: a toolbox for specialized analyses of microbial genomes and metagenomes. PeerJ Preprints https://peerj.com/preprints/1900/. Retrieved 1 November 2022.

[15] Stamatakis A. 2014. RAxML version 8: a tool for phylogenetic analysis and post-analysis of large phylogenies. Bioinformatics 30:1312–1313. doi:10.1093/bioinformatics/btu033. 24451623 PMC3998144

